# Human beta-defensin 1 circulating level and gene polymorphism in non-segmental vitiligo Egyptian patients^⋆^

**DOI:** 10.1016/j.abd.2022.04.002

**Published:** 2022-12-17

**Authors:** Azza Gaber Antar Farag, Mohamed Abd AlMoneam Shoeib, Azza Zagloul labeeb, Asmaa Shaaban Sleem, Hagar Mahmoud AbdElkader Khallaf, Amany Salah Khalifa, Mustafa Elsayed Elshaib, Nada Farag Elnaidany, Hayam Mohamed Aboelnasr Hanout

**Affiliations:** a Dermatology, Andrology and STDs Department, Faculty of Medicine Menoufia University, Shebin EL-koum, Egypt; b Microbiology and Immunology Department, Faculty of Medicine Menoufia University, Shebin EL-koum, Egypt; c Dermatology Department, Tala Central Hospital, Ministry of Health, Al-Menoufia, Egypt; d Clinical Pathology Department, Faculty of Medicine Menoufia University, Shebin EL-koum, Egypt; e Medical Student, Faculty of Medicine, Menoufia University, Shebin EL-koum, Egypt; f Clinical Pharmacy Department, Faculty of Pharmacy, MSA University, Egypt

**Keywords:** Genes, Polymorphism, genetic, Vitiligo

## Abstract

**Background:**

Vitiligo is an acquired depigmented skin disorder. It has a genetic and autoimmune background. Human beta defensin-1(HBD-1) plus its gene polymorphism were linked to some autoimmune disorders.

**Objective:**

To elucidate the possible role of HBD-1 in the pathogenesis of non-segmental vitiligo (NSV) through evaluation of HBD-1 serum levels and its single nucleotide polymorphism (SNP) in patients having NSV, in addition, to correlating the results with the extent of vitiligo in those patients.

**Methods:**

A current case-control study included 50 patients having NSV and 50 controls. The authors used Vitiligo Area Scoring Index (VASI) score to assess vitiligo severity and laboratory investigations to assess serum HBD-1 level using ELISA and defensin-beta1 (DEFB1) SNP using polymerase chain reaction-restriction fragment length polymorphism (PCR-RFLP).

**Results:**

There were significantly lower HBD-1 serum levels in NSV cases than in controls (p < 0.001). There was a significant predominance of GG DEFB1 genotype and G allele in NSV patients in comparison to controls (p < 0.001). The levels of serum HBD-1 and DEFB1 genotypes were not associated or correlated significantly with any of the personal and clinical parameters of vitiligo patients.

**Study limitation:**

The small sample size.

**Conclusions:**

DEFB1 gene polymorphism (GG genotype and G allele) may modulate vitiligo risk and contribute to vitiligo development in Egyptian populations. Decreased circulating HBD-1 levels might have an active role in vitiligo etiopathogenesis that could be mediated through its possible anti-inflammatory effects.

## Introduction

Vitiligo represents a developed defect in pigmentation. The main criterion of vitiligo is the melanocyte's loss from the epidermis and/or the loss of their function. Vitiligo is a quite common complaint, having a worldwide prevalence of 0.2%–1.8%.^1^ The vitiligo exact etiology ruins elusive, however, autoimmunity is supposed to play a vital role in its pathogenesis.^2^

Human Beta Defensins (HBDs) are small cationic peptides expressed in epithelial tissues all over the body.^3^ Eleven HBDs were identified.^4^ The first identified HBD was Human Beta Defensin (HBD-1) which was recognized in 1995.^5^ HBD's activate the innate immune responses having antimicrobial effects (antimicrobial peptides) against infection. Additionally, defensins have been concerned with development, immune modulation, and fertility as well as wound healing.^6^

Regarding their immune regulatory functions, defensins conglomerate both pro- and anti-inflammatory properties.^7^ The pro-inflammatory effects occur through defensin-receptor binding. Based on their catatonic nature, β-defensins interact with a diversity of receptors; that arise from electrostatic binding.^8^ The contradictory function of β-defensins (as anti-inflammatory) was demonstrated through their ability to attenuate a pro-inflammatory response.^9^

The mechanism through which β-defensins can counteract the pro-inflammatory reaction is not well identified, however, some mechanisms were considered. The binding of defensins (positively charged) to negatively charged ligands such as LPS is one possible mechanism that interferes with ligand binding. Additionally, defensins might act as antagonists for the receptors utilized by pro-inflammatory provocations. Moreover, β-defensins could induce the expression of some anti-inflammatory mediators. Furthermore, defensins (e.g., LL-37) may disrupt cell membranes inducing immune suppressive effects.^10^

HBD-1 is a 3928.6 Da peptide.^5^ It’s expressed principally in epithelia. It has an antimicrobial role against viruses plus gram-negative and positive bacteria.^11^ Besides this active antimicrobial function, HBD-1 has immunomodulator effects, as it is up-regulated in different inflammatory conditions.^12^ HBD-1 is programmed by the DEFB1 gene^13^ that mapped on chromosome 8p22.^14^

In view of autoimmunity, HBD-1 and DEFB1 gene polymorphisms were studied in some systemic and dermatological diseases with variable degrees of associations.^15^, ^16^, ^17^, ^18^ However, the association between this gene polymorphism and vitiligo has not been studied enough in different populations.^19^

Therefore, the authors aimed in this study to elucidate the possible role of HBD-1 in NSV pathogenesis through the evaluation of HBD-1 serum level and its gene polymorphism in a sample of Egyptian patients having NSV, in addition, to correlating the evaluated results with the clinical aspects of vitiligo in those patients.

## Patients and methods

The type of this study was a case control. It included 50 patients presented with NSV attending the Outpatient Clinic of Dermatology, Faculty of Medicine Menoufia University during the period from December 2019 to October 2020. Deﬁnite diagnosis of vitiligo based on the typical clinical presentation of the disease by two expert dermatologists.

The control group included 50 persons of gender and age-matched apparently healthy persons having no family history of vitiligo.

This study was approved by the Ethical Committee of Human Rights in Research at the Faculty of Medicine Menoufia University which was in accordance with the Helsinki Declaration in 1975 (revised in 2000). The study has an ethics committee approval number of (1202/2/4/20120).

Each participant received a complete explanation of the nature and purpose of the study. A written consent formula was got from every subject or his/her parent (<18 years) before the study initiation.

Patients having NSV from both sexes were included. Subjects having any of the following were excluded: 1) Systemic diseases e.g., diabetes mellitus, cirrhosis, infection, and renal failure. 2) Autoimmune (systemic or cutaneous) diseases (e.g rheumatoid arthritis and psoriasis).

The studied cases were subjected to history and clinical examination. A dermatological examination to identify the type of NSV and its distribution was done. VASI score was used to determine vitiligo severity.^20^

Five milliliters of venous blood were taken from each studied subject (patients and controls). Out of these 5 mL, 2 mL was left to clot and then centrifuged to separate serum. The separated sera were stored in a sterile plastic aliquot at −20 °C till the time of analysis for HBD-1 serum levels. The second part (3 mL) was stored at –20 °C in tubes holding Ethylene Diamine Tetra Acetic Acid (EDTA) for further examination of beta-defensin gene polymorphism by length polymorphism (PCR-RFLP).

### ELISA assay for serum beta-defensin-1 level

Serum beta-defensin-1 levels were measured by ELISA kits (NeoBioscience Technology Co., Ltd, Shenzhen, People’s Republic of China) regarding the instructions of the manufacturer.

### Genotyping for −20G/A (rs11362) DEFB1 gene polymorphism

Extraction of DNA was done using a blood sample through Gene JET™ Whole Blood Genomic DNA Purification Mini Kit (THERMO SCIENTIFIC, EU/Lithuania), following the manufacturer's instructions. SNP for −20G/A (rs11362) DEFB1 gene was performed by PCR-RFLP. Primer’s sequence was: F: CTT GAC TGT GGC ACC TCC CTT CAG-(sense) and R: -CAG CCC TGG GGA TGG GAA ACT C- (antisense). PCR reactions were carried out in a total volume of 30 uL containing 60 ng DNA, 3 µL 10 × PCR Gold Buffer, 2.5 mM MgCl2, 200 uM of each deoxynucleotide triphosphate, 0.4 mM of each primer, and 1 U of Ampli Taq Gold polymerase. Samples were denatured at 95 °C for 10 min followed by 30 cycles of 95 °C for the 60 s, 66 °C hybridization temperature for 60 s and 72 °C for the 60 s, and a final extension for 10 min at 72 °C. After PCR, the products were digested with a specific restriction enzyme, ScrFI (for G-20A) (Jingmei Biotech, Shanghai). Genotyping was performed blindly. The 268-bp PCR product was digested by ScrFI overnight at 37 °C.^21^

### Statistical analysis

Data were explored by the mean of Statistical Package for the Social Sciences (SPSS) version 23 and Epicalc 2000 programs. Statistics were divided into two parts: a) Descriptive statistics: e.g. mean (X¯), median, Standard Deviation (SD), range, Numbers(N), and percentages (%) and b) Analytic statistics using the Chi-Square test (χ2), Student *t* test (*t*), Mann-Whitney test (*U*), Kruskal Wallis test; p-value of was considered significant if it was ≤0.05.

## Results

The included 50 NSV patients, were 23 (46%) females and 27 (54%) males, the range of their age was 7‒60 years. There were non-significant differences between vitiligo patients and controls regarding their age (p = 0.335) and gender (p = 0.070) (Table 1).

**Table 1:** Personal and clinical data of the studied vitiligo patients and controls.

Personal characteristics	Vitiligo patients (n = 50)	Controls (n = 50)	Test of significance	p-value
**Age (years)**			U = 0.97	0.335
Mean ± SD	30.08 ± 14.46	32.30 ± 7.27		
Median	30	33		
Range	7‒60	18‒46		

	n	%	n	%	*χ*^2^	p-value
**Sex**					3.27	0.070
Males	27	54.0	18	36.0		
Females	23	46.0	32	64.0		
**Family history of vitiligo**						
Positive	6	12.0				
Negative	44	88.0	‒		‒	‒
**Disease duration/month**						
Mean ± SD	17.28 ± 17.78		‒		‒	‒
Median	12					
Range	3‒120					
**VASI score**						
Mean ± SD	3.72 ± 2.38		‒		‒	‒
Median	3.50					
Range	0.1‒10					
**Types of vitiligo**						
Acrofacial	18	36.0	‒		‒	‒
Generalized	10	20.0				
Focal	22	44.0				
**Hair affection**						
No	46	92.0	‒		‒	‒
Yes	4	8.0				
**Mucous membrane affection**						
No	46	92.0				
Yes	4	8.0	‒		‒	‒

U, Mann-Whitney test; *χ*^2^, Chi-Square test; n, number; VASI, Vitiligo Area Severity Index.

Out of these NSV patients, 6 cases of patients had a positive family history of vitiligo (6/50,12%). Disease duration ranged from 3‒120 months. The calculated VASI score ranged from 0.1 to 10. Regarding the type of vitiligo, 18 (36%) patients had acrofacial, 10 (20%) patients had generalized, and 22 (44%) had focal single patch vitiligo. Only 4 cases (8%) had leukotrichia and 4 cases (8%) had mucous membrane affection (Table 1).

### Serum HBD-1 levels

**Figure 1:**
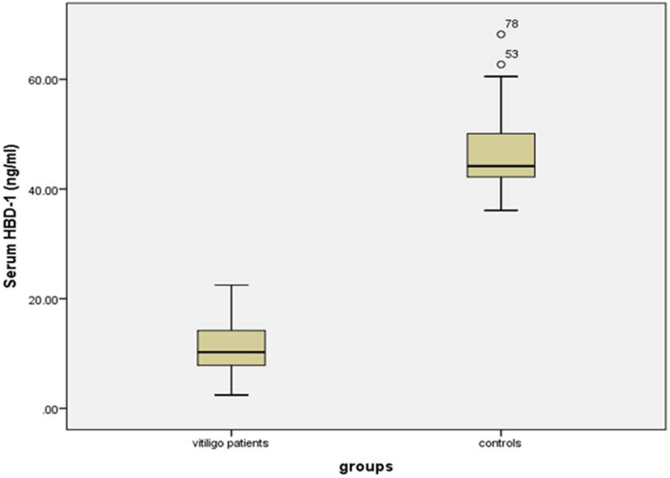
Serum HBD-1 levels in vitiligo patients and controls.

The investigated HBD-1 serum level was significantly low in vitiligo patients (11.14 ± 4.72 ng/mL) than in controls (46.53 ± 6.77 ng/mL) (p < 0.001) (Fig. 1).

### The relationship between HBD-1 serum levels and studied parameters of vitiligo patients

The levels of serum HBD-1 were not associated or correlated significantly with any personal or clinical data of vitiligo patients (p > 0.05 for all) (data not shown).

### Hardy-Weinberg equilibrium (HWE) analysis

Appling of HWE for DEFB-1 genotypes revealed that both cases and the control group had non-significant differences between observed and expected values (p = 0.290 and p = 0.432 respectively) (Table 2).

**Table 2:** Hardy-Weinberg equilibrium for DEFB-1 genotypes of vitiligo patients and control group.

DEFB-1 genotypes	Patients (n = 50)		Controls (n = 50)	
	Observed	Expected	Observed	Expected
GG	37	37.8	0	0.5
AG	13	11.3	10	9
AA	0	0.8	40	40.5
p-value	0.290		0.432	

HWE, Hardy-Weinberg equilibrium; n, number; DEFB-1, Defensin Beta-1.

### Distribution of DEFB-1 genotypes and alleles

**Figure 2:**
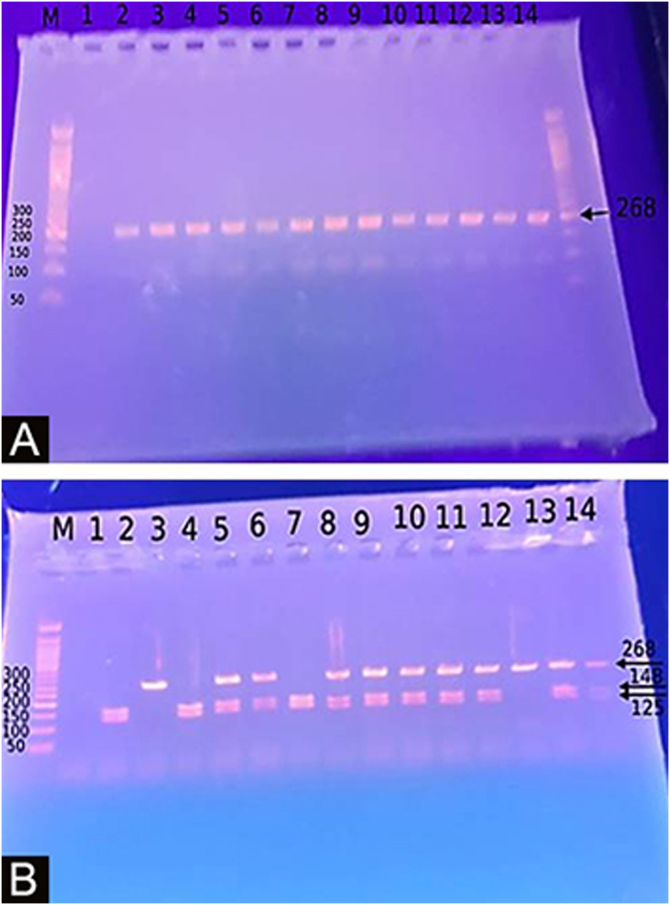
(A) Agarose gel electrophoresis images PCR product: 268-bp. (B) Agarose gel electrophoresis images for DEFB1 SNPs G20A, AA genotype: 268-bp, GG genotype: 143-bp, 125-bp, AG genotype.

The Study of the DEFB1 single nucleotide polymorphism (Fig. 2) showed that there was a significant predominance of GG genotype in vitiligo patients 37 (74%) and a predominance of the AA genotype in controls (p < 0.001). Also, the G allele was significantly demonstrated in studied cases 87 (87%) than in controls 10 (10%) increasing the risk of vitiligo by 60 times (p < 0.001; OR = 60.23) (Table 3).

**Table 3:** Percent distribution of DEFB-1 genotypes and alleles in vitiligo patients and control group.

DEFB-1 genotypes	Patients (n = 50)	Controls (n = 50)	*χ*^2^	p-value	OR (95% CI)
	n (%)	n (%)			
GG	37 (74.0)	0	77.39	< 0.001^a^	‒
AG	13 (26.0)	10 (20.0)			
AA	0	40 (80.0)			
**Alleles**	**(n = 100)**	**(n = 100)**	118.69	< 0.001^a^	60.23 (25.10‒144.56)
G	87 (87.0)	10 (10.0)			
A	13 (13.0)	90 (90.0)			

DEFB-1, Defensin Beta-1; OR, Odd's Ratio; CI, Confidence Interval; *χ*^2^, Chi-Square test; n, number.

a Significant.

### Relationship between HBD-1 serum level and its genotypes

The serum level of HBD-1 had a non-significant association with DEFB-1 genotypes in vitiligo patients (p = 0.611) or in the control group (p = 0.716) (Table 4).

**Table 4:** Serum HBD-1 level regarding defensin beta-1 genotypes in vitiligo patients and control group.

DEFB-1 genotypes	Serum HBD-1 levels (ng/mL)	
	Patients (n = 50)	Controls (n = 50)
	Mean ± SD	Mean ± SD
GG	10.82 ± 4.47	‒
AG	12.08 ± 5.44	45.23 ± 5.88
AA	‒	46.85 ± 7.00
Mann-Whitney test	0.51	0.36
p-value	0.611	0.716

HBD-1, Human Beta-Defensin-1; n, number; DEFB-1, Defensin Beta-1.

### Relationship between DEFB-1 genotypes and studied personal and clinical parameters of vitiligo patients

The DEFB-1 genotypes had a non-significant association with all studied personal and clinical data of vitiligo patients (p > 0.05 for all) (data not shown).

## Discussion

Th17 reaction is described by the elicitation of AMPs through IL-17A, IL-22, and IL-17F signaling, resulting in localized inflammation. AMPs including HBD-1, are able to chemoattract immature dendritic cells, T-cells and neutrophils directly via CCR6 signaling and indirectly through HBD-3 induction.^22^ In the existence of threat signals (such as oxidative stress and an extraordinary level of IL-6, IL-8 as well as heat shock protein 70) this chemoattraction could promote autoantigen presentation resulting in depigmentation.^23^, ^24^

Therefore, the authors expected up-regulation of circulating HBD-1 concentration in vitiligo patients than their matched peers. However, in this study, the authors observed significantly lower HBD-1 serum levels in vitiligo cases compared to controls.

Confirming this unexpected result, Ochoa-Ramírez et al.^25^ investigated 171 Mexican patients with NSV. They found that HBD-1 had lower estimated concentrations in patients with NSV than in controls. Moreover, the authors found that cases having active vitiligo demonstrated lower HBD-1 concentrations than those having stable disease, proposing that low circulating HBD-1 ranks are linked to vitiligo activity. Additionally, in Type 1Diabetes (T1D) (a CD8+ CTLs mediated disease), circulating HBD-1 levels were reported to be significantly lower than in the control group.^16^, ^17^, ^18^

Regarding T1D, a possible clarification for that result is that the extreme CD8+ cytotoxic T-cell subgroup activation, characteristic of T1D, negatively affects HBD-1.^26^ Additionally, insulin signaling is important for HBD-1 ideal expression through increasing intracellular glucose concentrations and mediating gene expression.^27^

However, in vitiligo, the authors suggested that dermal CD8+ CTL subpopulation might be responsible for local production and local up-regulation of HBD-1 that participate in local tissue up-regulated inflammatory process and depigmentation without any systemic effect on HBD-1 levels. Also, the authors suggested that HBD-1 might be shifted from bloodstream to vitiligous skin inducing depigmentation, and this shift resulted in its lower serum levels.

Confirming the present hypothesis regarding HBD-1 local inflammatory effects, Polesello et al.^18^ and Ozlu et al.^19^ demonstrated an increase in HBD-1 in saliva and in skin biopsies in oral lichen planus and psoriasis respectively. Therefore, studies to assess both systemic and tissue HBD-1 levels simultaneously are recommended.

Another explanation for the current demonstrated low HBD-1 level in vitiligo cases could be that HBDs offer a systemic anti-inflammatory function.^10^ Recently, it was hypothesized that HBD-2 could suppress dendritic cell-mediated secretions of pro-inflammatory cytokines such as IL-1β, IL-12, and TNF-α in Inflammatory Bowel Disease (IBD)^28^ as well as decrease IL-6 and TNF-α in lung tissues.^29^ Also, HBD-3 reduces the secretion of IL-6 and IL-8, showing hopeful potential as adjuvant therapy for the treatment of inflammatory periodontitis.^9^

Thus, the authors hypothesized that in vitiligo, HBD-1 could act as an anti-inflammatory peptide, and the current demonstrated low HBD-1 serum levels in vitiligo patients may be translated into a repressed anti-inflammatory activity. Therefore, further studies on HBD-1 are required to verify this hypothesis.

In the current study, the authors found that serum HBD-1 was not affected by any evaluated personal or clinical data of vitiligo patients. This result was in agreement with that of Ochoa-Ramírez et al.^25^ who observed a non-significant association between serum HBD-1 levels and clinical characteristics of vitiligo.

The DEFB1 gene (located in chromosome 8p22) consists of two exons, the first encodes the leucine-rich pro sequence and signal. The second exon however encodes the mature peptide.^7^ SNPs of that gene could occur at different sites of the first exon’s 50 noncoding regions,^26^ including −52G>A (rs1799946), −44C>G (rs1800972), and −20G>A (rs11362).^18^

In the current work, the authors analyze −20G/A (rs11362) DEFB1 genotypes polymorphism. The authors found that there was a significant predominance of GG DEFB1 (−20G/A) genotype in vitiligo patients than controls, as well as the G allele which increased the possibility of vitiligo occurrence by about 60 times. However, in controls, the authors demonstrated that DEFB1 (−20G/A) AA genotype and A allele were significantly frequent and were considered of protecting value.

In agreement with this result, Ochoa-Ramírez et al.^25^ observed that there was a predominance of GG genotype at position-20 in vitiligo patients than controls. Also, Salem et al.^30^ studied 50 Egyptian NSV patients and revealed that the DEFB1 (−20G/A) AA genotype and A allele had significantly lower frequencies in vitiligo patients and exerted a protective effect against vitiligo development.

Additionally, in atopic dermatitis (a T-cell-mediated inflammatory disease), de Oca et al.^31^ found that the −20GG genotype is a genetic risk issue for atopic dermatitis development. Moreover, in IBD (an immune-inflammatory disease), Zanin et al.^32^ reported that IBD patients had more frequent G alleles more frequent than controls. Furthermore, in SLE (an autoimmune disease), Sandrin et al.^33^ reported that the AA genotype and its A allele, were of less significant frequencies in the patient group compared to the control, showing protective effects.

Certain polymorphisms in DEFB1, might affect DEFB1 transcription activity and consequently HBD-1 protein expression. Actually, polymorphisms in DEFB1 50 untranslated region alter the putative transcription factor binding site for the nuclear factor-KB p105 subunit resulting in HBD1 protein expression.^18^

In the current study, serum HBD-1 levels were not significantly affected by DEFB1 SNP either in vitiligo patients or in the control group. Supporting this result, Ochoa-Ramírez et al.^25^ observed a non-significant association between serum HBD-1 concentrations and DEFB1 genotypes.

However, in lBD Zanin et al.^33^ reported that colonic Crohn’s Disease localization was linked with impaired expression of HBD-1. As the (c.−20G/A) A allele seems to be related to local reduced HBD-1 expression levels. The authors concluded that DEFB1 polymorphism may cause lower expression of HBD-1 in colonic epithelial cells. The different pathogenic mechanisms of vitiligo and Crohn’s disease as well as a different sample size in their (n = 145) and the present study (n = 50) could explain the difference.

In this study, the authors observed that the DEFB1 genotypes had no significant effects on any of the studied personal characteristics or clinical data of the studied vitiligo patients (age, sex, duration of disease, and VASI). Confirming this study, Ochoa-Ramírez et al.^25^ observed a non-significant association between DEFB1 genotypes and the studied clinical data of NSV cases.

However, Salem et al.^30^ the study was in partial agreement with these results. They demonstrated an insignificant difference in DEFB1 (−20G/A) genotype distribution in relation to different history and clinical findings except for the mean VASI score. They found that AA genotype carriers were associated with significantly lower VASI scores. This difference might be due to the small sample size in each study (n = 50 NSV patients) and/or different selection criteria of the investigated cases as they studied only patients with active NSV while the authors studied patients with NSV regardless of disease activity.

The study limitations were a) The low number of investigated cases, b) Its structure (a case-control study) and c) It evaluated only a single inflammatory marker rather than multiple ones.

## Conclusions

It seems that DEFB1 gene polymorphism at -20 might modulate vitiligo development risk as the DEFB1 (−20G/A) GG genotype and G allele contribute to vitiligo development in Egyptian populations. Decreased circulating HBD-1 levels might have an active role in vitiligo etiopathogenesis that could be mediated through its possible anti-inflammatory effects.

## Financial support

None declared.

## Authors' contributions

All authors should have made substantial contributions to all of the following.

Azza Gaber Antar Farag: Critical literature review; study conception and planning; approved the final article.

Mohamed Abd Al Moneam Shoaib: Study conception and planning; approved the final article.

Azza Zagloul labeeb: Data collection, analysis, and interpretation; approved the final article.

Asmaa Shaaban Sleem: Data interpretation; approved the final article.

Hagar Mahmoud AbdElkader Khallaf: Data collection; approved the final article.

Amany Salah Khalaf: Data analysis and interpretation; approved the final article.

Mustafa Elsayed Elshaib: Statistical analysis; approved the final article.

Nada Farag Elnaidany: Statistical analysis; approved the final article.

Hayam Mohamed Aboelnasr Hanout: Study planning; approved the final article.

## Conflicts of interest

None declared.
